# Development, Relative Validity, and Reproducibility of a Short Food Frequency Questionnaire for the Japanese

**DOI:** 10.3390/nu14204394

**Published:** 2022-10-20

**Authors:** Akiko Nanri, Aya Fujiwara, Haruka Miyake, Ikuko Kashino, Tetsuya Mizoue

**Affiliations:** 1 Department of Food and Health Sciences, International College of Arts and Sciences, Fukuoka Women’s University, Fukuoka 813-8529, Japan; 2 Department of Epidemiology and Prevention, Center for Clinical Sciences, National Center for Global Health and Medicine, Tokyo 162-8655, Japan

**Keywords:** food frequency questionnaire, Japanese, reproducibility, validity

## Abstract

We have developed a short food frequency questionnaire (FFQ) for use in assessing diet quality for Japan, with special reference to the prevention of non-communicable diseases. Here, we assessed the ranking performance of this FFQ and its reproducibility. We developed a 28-item (21 food groups and 7 beverage) FFQ with consideration to both Japanese dietary culture and evidence of disease prevention. Twenty-four university faculty members participated in the validation study. They completed 3-day photographic food record and answered the FFQ on the next day of the last food record (time 1) and a week later (time 2). We calculated Spearman correlation coefficients between intakes of food groups from photographic food records and the consumption frequency from the FFQs (ranking ability) and between the consumption frequency of food groups from the FFQs (time 1 and time 2) (reproducibility). Spearman correlation coefficients between the food records and FFQ (time 1) ranged from −0.12 to 0.86 (median 0.51). These values were comparable to those in comparison with FFQ (time 2). After energy adjustment of intakes from the food records, the corresponding values were somewhat weakened for many food groups. The correlation coefficients between two FFQs ranged from 0.14 to 0.96 (median 0.79). The short FFQ showed acceptable reproducibility and ability to rank the consumption of most food groups.

## 1. Introduction

Diet plays an important role in the prevention of non-communicable diseases [1]. In nutritional epidemiology, the quality of diet and dietary patterns, a comprehensive variable that integrates consumption of several foods or food groups, has attracted substantial interest [2]. In the Japan Public Health Center-based Prospective (JPHC) study, closer adherence to the Japanese dietary guidelines [3] was associated with decreased risk of all-cause and cardiovascular disease mortality [4], and a prudent dietary pattern—characterized by high intake of vegetables, fruit, soy products, potatoes, seaweed, mushrooms, and fish—was associated with decreased risk of death from all causes, cardiovascular disease, and suicide [5,6]. These findings highlight the need to evaluate diet quality in efforts to prevent non-communicable disease and premature death.

Food frequency questionnaires (FFQs) are frequently used to assess diet in epidemiologic studies and normally include queries on the intake of over 100 food and beverage items [7,8], requiring 30–60 min to complete [9]. The use of such FFQs is therefore not feasible in time-limited settings (i.e., health examinations and clinics). For ranking of intake at the food group level, a shorter FFQ may perform well while reducing the burden on respondents. If so, such tools would be useful not only for nutrition research (i.e., dietary patterns) but also for self-assessment of the usual diet.

In Japan, several short FFQs comprising 10 to 30 food or beverage items have been developed [10,11,12,13,14]. Yatsuya et al. [14] developed a nine food-item questionnaire and reported that the correlation coefficients for reproducibility ranged between 0.37 and 0.86 and those for the validity between 0.01 and 0.56. Sauvaget et al. [12] showed the correlation coefficients for the validity of 22 food items ranged between −0.03 and 0.51. However, these tools do not differentiate foods that have been linked to lower or no increased risk of diseases (chicken, whole grains, etc.) from foods that have been linked to increased risk of diseases (red meat and processed meat, refined grains, etc.) [10,11,12,13,14]. Moreover, they do not include specific foods typical to the Japanese diet, such as mushrooms, seaweeds, and green tea [10,11,12,13,14]. For these reasons, the use of these FFQs may be limited in the analysis of diet quality and dietary pattern.

To address these issues, we developed a short FFQ for Japanese people to assess the consumption frequency of foods and food groups, as part of the Japan Epidemiology Collaboration of Occupational Health (J-ECOH) Study, an ongoing, large-scale study of workers in Japan. Here, we describe the process of questionnaire development and the ranking performance and reproducibility of this FFQ.

## 2. Materials and Methods

### 2.1. Development of Short Food Frequency Questionnaire

After reviewing existing FFQs [8,15] and previous dietary pattern analyses [16,17] in Japan, we selected food groups and beverages for inclusion in the FFQ in accordance with epidemiological evidence and/or Japanese dietary culture: for instance, whole grains, poultry, and coffee for decreased risk of type 2 diabetes, hypertension, depression, cardiovascular disease, total cancer, and all-cause mortality [18,19,20,21,22,23,24,25]; red meat and processed meat, sugar- and artificially sweetened beverages, salty foods, and fried foods for increased risk of type 2 diabetes, and all-cause, cardiovascular disease, and cancer mortality [25,26,27,28,29,30,31]. In addition, miso soup, soy products, mushrooms, seaweeds, and green tea are typical foods/beverages in the Japanese diet and have been linked to favorable health effects [32,33].

Finally, we created a 28-item FFQ, which comprised 21 food groups (rice with barley and millet, brown rice, and rice with germ; white rice; whole-grain bread, rye bread, barley bread, and millet bread; other breads such as white bread and sweet buns; noodles; potatoes; miso soup; soy products; raw vegetables; cooked vegetables; mushrooms; seaweeds; fruits; fish and shellfish excluding dried fish and salty fish; beef, pork, liver, and processed meat; chicken; eggs; milk and dairy products; nuts; salty foods such as pickled plums, pickled vegetables, dried fish, salty fish, and fish roe; and fried foods such as tempura, fried chicken, deep-fried foods, cutlets, and French fries) and 7 beverage items (coffee; green tea; other tea such as black tea, oolong tea, and blend tea; water; 100% fruit and vegetable juice; sugar-sweetened beverages including soft drink, coffee, black tea; and artificially sweetened beverages such as non-caloric and low-caloric beverages). For each item, we prepared 8 options regarding consumption frequency over the past month (almost never, 1–3 times/month, 1–2 times/week, 3–4 times/week, 5–6 times/week, 1 time/day, 2 times/day, or ≥3 times/day for food groups; and almost never, 1–3 cups/week, 4–6 cups/week, 1 cup/day, 2 cups/day, 3 cups/day, 4 cups/day, ≥5 cups/day for beverage items). The frequency options were determined with reference to existing Japanese FFQs [8,15].

### 2.2. Study Procedure and Participants

**Figure 1:**
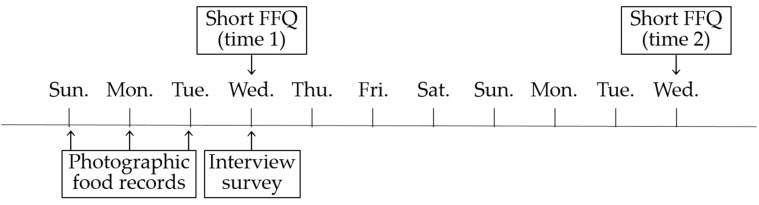
Schedule of the validation study.

The present validation study was conducted among the faculty members of Fukuoka Women’s University. Due to the limited resources, we set a maximum number of participants of 30. We recruited participants through posters and e-mail from October to November 2018. As a result, 24 persons (9 males and 15 females, aged 23–64 years) agreed to participate. The validation study was scheduled as illustrated in Figure 1. Participants were asked to take a picture of everything they ate and drank for three consecutive days from 25 November 2018 to 26 February 2019 (photographic food records), and bring them to a laboratory for confirmation of what and how much they consumed in the day following the last day of photographic food recording (interview survey). The number of days for photographic food records was determined to cover the diet on both weekday and weekend while minimizing the burden on the participants. Participants were also asked to answer the short FFQ we developed twice (time 1 and time 2). First, participants responded to the paper-based FFQ (time 1) in the laboratory before the interview survey. They then received FFQ (time 2) as an e-mail attachment one week later, and submitted their completed FFQ (time 2) by e-mail or by hand of the printed-out paper version. We also used the FFQ to collect data on the participants’ age, sex, height, and weight. The study protocol was approved by the Ethics Committee of Fukuoka Women’s University (ethical approval number 2018–20), and written informed consent was obtained from all participants prior to the survey.

### 2.3. Photographic Food Records and Interview Survey

Prior to the survey, participants received a digital camera and were instructed to take photos of all dishes, foods, and beverages they consumed during three consecutive days from Sunday to Tuesday. We asked participants to take a photo from above the dish before eating and another photo of any leftover food. On Wednesday, the participants visited our laboratory for an interview survey to confirm their photo-recorded diet with reference to food models. These collected data were reviewed and coded according to the Standard Tables of Food Composition Japan 2020 [34] by one of the authors (A.N.), a dietician. We obtained 407 food codes from the photographic food records. Of these, 281 food codes were classified into the 27 food groups and beverages excluding artificially sweetened beverage in the FFQ. Since artificially sweetened beverage was not included in the Standard Tables of Food Composition Japan, artificially sweetened beverages consumed during the 3-day photographic food record were classified as an artificially sweetened beverage for the FFQ, without a food code. (Only one participant consumed an artificially sweetened beverage during the survey.) Miso soup and fried foods were classified not only as their corresponding food groups but also as other food groups according to their ingredients. The amount of leftover food, if any, was subtracted from the intake estimated from the photographic food record to obtain actual intake. We then calculated the average daily intake for each food group by dividing their total intake over the three days of dietary records by three. Food group intakes were adjusted for energy by the residual method [35]. The energy-adjusted intake of each participant was calculated as the sum of the residual for each participant from a regression model with food intake as the dependent variable and total energy intake as the independent variable and the expected food intake for a person with mean energy intake [36]. The photographic food record can precisely estimate dietary intake. Matsushita et al. [37] observed that the correlation coefficients between the estimated food intake from the 24 h recall combined with a portable camera and weighed food intake were 0.7 or higher in most food groups. Saeki et al. [38] reported that the rank correlation coefficients between estimated intake from the food-photographic record and weighed intake ranged from 0.78 to 0.94 for the 44 nutrients.

### 2.4. Statistical Analysis

Characteristics of the study participants are expressed as means (standard deviations), median, and range for age and BMI according to sex. First, we assigned the selected frequency category in the short FFQ the following values to estimate daily frequency of consumption: almost never, 0; 1–3 times/month, 0.07; 1–2 times/week, 0.21; 3–4 times/week, 0.5; 5–6 times/week, 0.79; 1 time/day, 1; 2 times/day, 2; and ≥3 times/day, 3 for food groups and almost never, 0; 1–3 cups/week, 0.29; 4–6 cups/week, 0.71; 1 cup/day, 1; 2 cups/day, 2; 3 cups/day, 3; 4 cups/day, 4; and ≥5 cups/day, 5 for beverage items. Second, we calculated the means (standard deviations) and median of crude intake and energy-adjusted intake for each food group estimated from the photographic food records and consumption frequency assessed from the short FFQs. Energy, protein, fat, and carbohydrate intake from the photographic food records was estimated using the Standard Tables of Food Composition Japan 2020 [34].

Our short FFQ was designed to capture the intake frequency of each food group to assess dietary pattern, rather than to estimate the intake of each nutrient, food, or energy. Therefore, for preciseness of ranking (validity), we calculated the Spearman correlation coefficient between crude intake or energy-adjusted intake derived from the 3-day photographic food record and consumption frequency derived from the FFQs. Regarding reproducibility, Spearman correlation coefficients between the consumption frequency of the 28 food groups and beverages from the two FFQs one week apart were calculated. Previous validation studies have typically correlation coefficients of 0.5 to 0.7 [35]. In addition, regarding validated dietary questionnaires in Japan, median correlation coefficients between dietary records and FFQs (validity) from 0.31 to 0.56, and those between the same FFQ completed at two time points (reproducibility) have ranged from 0.50 to 0.72 [8]. We therefore defined a correlation coefficient of ≥0.31 for validity and ≥0.50 for reproducibility as acceptable. When we presented these results, we sorted the food groups in the same order as in the Standard Tables of Food Composition Japan 2020 [34]. All analyses were performed using Statistical Analysis System (SAS) version 9.4 (SAS Institute, Cary, NC, USA).

## 3. Results

All 24 males and females who participated in the present study completed the 3-day photographic food records and two FFQs (time 1 and time 2) (i.e., no dropouts). Regarding participant characteristics, mean ± standard deviation (median, range) of age and BMI in males and females were 39.3 ± 11.9 (34, 25–64) and 37.0 ± 8.3 (35, 23–61) years, respectively, and 22.1 ± 4.0 (21.4, 15.8–29.7) and 20.3 ± 2.1 (19.7, 18.0–25.9) kg/m^2^, respectively. Mean (standard deviation) intakes of total energy, protein, fat, and carbohydrate estimated using 3-day photographic food record in males and females were 2103 (490) and 1608 (346) kcal/day for total energy intake, 12.7 (1.8) and 13.7 (1.7) %energy for protein, 33.9 (9.4) and 32.0 (5.2) %energy for fat, and 45.6 (9.1) and 49.8 (5.8) %energy for carbohydrate. Mean and median consumption frequencies of food groups based on the two short FFQs and food group intakes based on the 3-day photographic food record are shown in Table 1. Mean consumption frequency of food groups based on FFQ (time 1) were comparable with those based on FFQ (time 2).

### 3.1. Ranking Performance

Spearman correlation coefficients between consumption frequency of food groups based on the two FFQs and food group intakes based on the 3-day photographic food record are shown in Table 2. Spearman correlation coefficients between crude intake based on the 3-day photographic food record and consumption frequency based on FFQ (time 1) ranged from −0.12 for beef, pork, liver, and processed meat to 0.86 for fruits (median 0.51). The correlation coefficients were ≥0.31 for 25 items excluding potatoes (r = 0.10), cooked vegetables (r = 0.11), and beef, pork, liver, and processed meat (r = −0.12). The correlations between photographic food records and FFQ (time 2) were comparable to those between photographic food records and FFQ (time 1). For cooked vegetables and beef, pork, liver, and processed meat, although correlations between the photographic food records and FFQ (time 1) were low, the corresponding values between the photographic food records and FFQ (time 2) were somewhat improved (r = 0.24 for cooked vegetables and 0.25 for beef, pork, liver, and processed meat). In contrast, correlations were low in the intake of potatoes in both comparisons between photographic food records and FFQ (time 1) (r = 0.10) or FFQ (time 2) (r = −0.05). After energy adjustment of intakes estimated by the photographic food records, the correlation coefficients between photographic food records and FFQ (time 1) or FFQ (time 2) were somewhat weakened in the intakes of many but not all food groups.

### 3.2. Reproducibility

Spearman correlation coefficients between the two FFQs ranged from 0.14 for beef, pork, liver, and processed meat to 0.96 for coffee (median 0.79). Correlation coefficients between the two FFQs of 26 food groups excluding beef, pork, liver, and processed meat (r = 0.14) and salty foods (r = 0.40) were ≥0.50. In particular, corresponding values of rice with barley and millet, brown rice, rice with germ, whole-grain bread, rye bread, barley bread, other bread, miso soup, raw vegetables, fruits, coffee, and green tea were >0.90.

## 4. Discussion

We developed a short FFQ consisting of 28 food and beverage items to assess the diet over the past month and assessed the ranking performance of the FFQ using 3-day photographic food record and its reproducibility of the FFQ in two administrations given one week apart. In the present validation study, we observed acceptable ability to rank the consumption frequency of food groups derived from FFQ and reproducibility of the FFQ. Spearman correlation coefficients between intakes from the photographic food records and consumption frequency from FFQ (time 1) ranged from −0.12 to 0.86 (median 0.51) and were ≥0.31 for 25 food groups. The corresponding values for consumption frequency between two administrations of the FFQ ranged from 0.14 to 0.96 (median 0.79) and were ≥0.50 for 26 food groups.

The ranking performance of FFQ was acceptable for most food items. The median correlation coefficient between the photographic food record (amount of consumption) and FFQ (time 1) (consumption frequency) was comparable to those between intakes from food records and intakes from FFQs (food and beverage items: 9 to 138) developed previously in Japan, which ranged from 0.22 to 0.51 [14,39,40,41,42,43,44,45,46]. Ranking performance of the consumption frequency of potatoes based on FFQ (time 1) and FFQ (time 2) was low in the present study (0.10 for FFQ (time 1) and −0.05 for FFQ (time 2)). Some previous studies have observed similar results (correlation coefficients of potatoes for validity: 0.09 to 0.21 [44,45,47,48]. In Japan, potatoes are often eaten as a garnish or in combination with other foods, and the amount of intake per time ranges widely. It may be therefore difficult for participants to report their habitual consumption frequency of potatoes using the FFQ. Of foods with scientific evidence of disease prevention, the ranking performance was the highest for fruits (r = 0.86), followed by coffee (r = 0.77), green tea (r = 0.57), milk and dairy products (r = 0.55), and whole grains (r = 0.53). Of foods with evidence of high disease risk, the ranking performance was the highest for artificially sweetened beverages (r = 0.53), followed by sugar-sweetened beverages (r = 0.50), white bread (r = 0.50), and fried foods (r = 0.44).

We observed acceptable reproducibility of the consumption frequency of food groups based on the FFQ. Among short FFQs including relatively small numbers of food and beverage items (<50 items) developed in Japan, Yatsuya et al. [14] reported a median (range) correlation coefficient of consumption frequency of nine food groups derived from two food intake and behavior checklists (consisting of nine food items) administered over a 9-month interval of 0.60 (0.37–0.86). Ogawa et al. [49] observed corresponding values of intakes of 40 food items based on two FFQs (consist of 40 food items) over a 1-year interval of 0.43 (0.14–0.76) in males and 0.45 (0.06–0.74) in females. Imaeda et al. [39] reported that values of 20 food group intakes based on two FFQs (consisting of 47 food items) over 1-year interval of 0.61 (0.38–0.86) in males and 0.66 (0.45–0.84) in females. In the JPHC Study, median (range) correlation coefficients of 17 food group intakes assessed using a 44-item FFQ at a 5-year interval were 0.34 (0.15–0.63) in males and 0.48 (0.18–0.55) in females [40]. In most of these previous studies, the interval between FFQs was from 9 months to 1 year and the median of correlation coefficients ranged from 0.34 and 0.66. In the present study, reproducibility of the FFQ was somewhat higher (median correlation coefficient, 0.79) than in the previous studies, which may be due to the small number of food items contained in the FFQ and short interval between the two administrations. If the interval between two FFQs is short, participants may remember their previous responses [35]. Of foods with scientific evidence of disease prevention, the reproducibility was the highest for coffee (r = 0.96), followed by green tea (r = 0.94), fruits (r = 0.92), and whole-grain bread (r = 0.92). Of foods with evidence of high disease risk, the reproducibility was the highest for white bread (r = 0.91), followed by sugar-sweetened beverages (r = 0.81), fried foods (r = 0.72), and artificially sweetened beverages (r = 0.71).

Our study had some limitations. First, participants in this validation study might have been more health conscious than nonparticipants. Therefore, the present findings might not be applicable to the entire population of the J-ECOH Study. Second, we determined the sample size mainly by feasibility such as research resources. Due to the small number of participants, we cannot examine the validity and reproducibility according to background factors. Third, the photographic food records we used to minimize the burden on the participants were not dietary records that recorded the intake of all foods, dishes, and drinks that participant ate. In addition, the photographic food record was only three days’ duration. Accordingly, we might not have been able to accurately assess the participant’s usual dietary intake. Fourth, the location where the FFQ was completed differed between the test (laboratory) and the retest (arbitrary). We cannot exclude a possibility of some influence of this difference on the reproducibility. Fifth, the data coding of the photographic food records was performed by one dietician. Finally, as mentioned above, to reduce the burden on the participants and obtain their cooperation, we conducted FFQ (time 1) on the day of the interview survey (day following the 3-day photographic food record). Since participants’ recall of dietary intake in answering FFQ (time 1) might have been influenced by administration of the photographic food records, ranking performance (correlation coefficients between the photographic food record and FFQ (time 1)) might be higher than the true value.

## 5. Conclusions

In conclusion, this 28-item FFQ showed acceptable ranking performance and reproducibility for many food groups. The present data would be helpful in the interpretation of the results of epidemiological studies using this questionnaire. Validation study in a different sample will ensure the validity of this questionnaire for studies in various settings.

## Figures and Tables

**Table 1:** Consumption frequency of food groups based on two FFQs one week apart and food group intakes based on a 3-day photographic food record in 9 males and 15 females.

	FFQ (Time 1) (Times or Cups/Day)		FFQ (Time 2) (Times or Cups/Day)		Photographic Food Records (g/Day)			
					Crude		Energy-Adjusted ^1^	
	Mean ± SD	Median	Mean ± SD	Median	Mean ± SD	Median	Mean ± SD	Median
Foods or Food groups								
Rice with barley and millets, brown rice, rice with germ	0.3 ± 0.7	0.1	0.3 ± 0.7	0.0	33 ± 107	0	33 ± 106	0
White rice	1.4 ± 0.9	2.0	1.2 ± 0.9	0.9	233 ± 122	244	246 ± 172	208
Whole-grain bread, rye bread, barley bread, millet bread	0.1 ± 0.2	0.0	0.1 ± 0.2	0.0	5 ± 18	0	4 ± 11	0
Other bread (white bread, etc.)	0.4 ± 0.4	0.2	0.5 ± 0.4	0.4	36 ± 31	36	39 ± 38	33
Noodles	0.3 ± 0.2	0.2	0.3 ± 0.2	0.2	79 ± 81	70	95 ± 147	65
Potatoes	0.2 ± 0.2	0.2	0.3 ± 0.2	0.2	28 ± 32	11	27 ± 29	11
Soy products	0.5 ± 0.4	0.5	0.5 ± 0.5	0.4	52 ± 68	31	72 ± 123	22
Nuts	0.2 ± 0.3	0.0	0.2 ± 0.3	0.0	2 ± 6	0	2 ± 6	0
Raw vegetables	0.6 ± 0.4	0.5	0.6 ± 0.4	0.5	64 ± 72	35	64 ± 82	36
Cooked vegetables	0.9 ± 0.6	0.8	0.8 ± 0.5	0.8	127 ± 53	120	126 ± 49	128
Fruits	0.4 ± 0.4	0.2	0.4 ± 0.5	0.1	55 ± 68	33	60 ± 82	17
Mushrooms	0.3 ± 0.3	0.2	0.3 ± 0.2	0.2	10 ± 14	6	10 ± 14	5
Seaweeds	0.3 ± 0.2	0.2	0.2 ± 0.2	0.1	4 ± 4	2	4 ± 5	3
Fish and shellfish (excluding dried fish and salty fish)	0.3 ± 0.2	0.2	0.3 ± 0.3	0.2	46 ± 41	40	47 ± 42	34
Beef, pork, liver, processed meat	0.6 ± 0.4	0.5	0.5 ± 0.3	0.5	68 ± 34	67	66 ± 31	64
Chicken	0.5 ± 0.3	0.5	0.6 ± 0.4	0.5	43 ± 39	39	50 ± 65	32
Eggs	0.5 ± 0.4	0.4	0.5 ± 0.4	0.2	35 ± 25	33	35 ± 26	31
Milk, dairy products	0.6 ± 0.5	0.6	0.6 ± 0.5	0.5	93 ± 61	91	96 ± 70	74
Miso soup	0.5 ± 0.6	0.2	0.5 ± 0.5	0.2	54 ± 62	46	83 ± 189	10
Salty foods (pickled vegetables, dried fish, salty fish, etc.)	0.2 ± 0.3	0.1	0.3 ± 0.4	0.2	17 ± 15	13	16 ± 15	12
Fried foods	0.3 ± 0.2	0.2	0.4 ± 0.3	0.2	66 ± 51	64	64 ± 79	45
Beverages								
Green tea	1.9 ± 1.7	2.0	1.8 ± 1.8	1.0	221 ± 215	167	219 ± 211	208
Other tea (black tea, oolong tea, etc.)	1.8 ± 1.7	2.0	1.5 ± 1.5	0.9	348 ± 355	265	354 ± 351	352
Coffee	1.6 ± 1.3	2.0	1.6 ± 1.1	2.0	159 ± 152	108	158 ± 152	96
Water	1.5 ± 1.8	0.9	1.7 ± 1.7	1.0	329 ± 360	200	579 ± 1256	105
100% Fruit and vegetable juice	0.2 ± 0.3	0.0	0.2 ± 0.3	0.0	16 ± 35	0	19 ± 54	0
Sugar-sweetened beverages	0.3 ± 0.7	0.0	0.3 ± 0.7	0.0	80 ± 154	0	84 ± 197	0
Artificially sweetened beverages	0.1 ± 0.4	0.0	0.0 ± 0.1	0.0	5 ± 24	0	4 ± 18	0

^1^ Food group intakes based on photographic food records were adjusted for energy intake by the residual method.

**Table 2:** Spearman correlation coefficients between consumption frequency of food groups based on two FFQs one week apart and food group intakes based on a 3-day photographic food record in 9 males and 15 females.

	FFQ (Time 1) vs. FFQ (Time 2)	Photographic Food Records vs. FFQ (Time 1)		Photographic Food Records vs. FFQ (Time 2)	
		Crude	Energy-Adjusted ^1^	Crude	Energy-Adjusted ^1^
Foods or Food groups					
Rice with barley and millets, brown rice, rice with germ	0.90	0.53	0.34	0.53	0.32
White rice	0.69	0.33	0.55	0.47	0.37
Whole-grain bread, rye bread, barley bread, millet bread	0.92	0.52	0.35	0.47	0.31
Other bread (white bread, etc.)	0.91	0.50	0.46	0.66	0.65
Noodles	0.72	0.52	0.48	0.69	0.50
Potatoes	0.77	0.10	0.03	−0.05	−0.13
Soy products	0.81	0.47	0.32	0.45	0.30
Nuts	0.84	0.32	0.15	0.41	0.17
Raw vegetables	0.91	0.50	0.28	0.57	0.24
Cooked vegetables	0.72	0.11	0.18	0.24	0.22
Fruits	0.92	0.86	0.82	0.77	0.72
Mushrooms	0.62	0.52	0.53	0.34	0.45
Seaweeds	0.77	0.61	0.58	0.71	0.69
Fish and shellfish (excluding dried fish and salty fish)	0.80	0.42	0.32	0.44	0.38
Beef, pork, liver, processed meat	0.14	−0.12	−0.01	0.25	0.21
Chicken	0.59	0.31	0.36	0.44	0.39
Eggs	0.68	0.55	0.49	0.48	0.48
Milk, dairy products	0.83	0.55	0.37	0.56	0.57
Miso soup	0.95	0.72	0.74	0.73	0.72
Salty foods (pickled vegetables, dried fish, salty fish, etc.)	0.40	0.43	0.48	0.76	0.62
Fried foods	0.72	0.44	0.20	0.35	−0.03
Beverages					
Green tea	0.94	0.57	0.53	0.48	0.46
Other tea (black tea, oolong tea, etc.)	0.73	0.50	0.57	0.41	0.43
Coffee	0.96	0.77	0.68	0.79	0.75
Water	0.78	0.73	0.63	0.78	0.52
100% Fruit and vegetable juice	0.86	0.67	0.50	0.53	0.45
Sugar-sweetened beverages	0.81	0.50	0.43	0.66	0.53
Artificially sweetened beverages	0.71	0.53	0.48	0.69	0.46

^1^ Food group intakes based on photographic food records were adjusted for energy intake by the residual method.

## Data Availability

The data presented in this study are available on request from the corresponding author.
